# Supplementary material for: Force-velocity profiling in athletes: Reliability and agreement across methods
Source: PLoS One. 2021 Feb 1;16(2):e0245791. doi: 10.1371/journal.pone.0245791 (PMC7850492; doi:10.1371/journal.pone.0245791)
Supplement: Attachment — Submitted filename: Valenzuela (2020) Reliability fv parameters.pdf [file pone.0245791.s002.pdf]

International Journal of Sports Physiology and Performance, (Ahead of Print)
https://doi.org/10.1123/ijspp.2020-0308
© 2020 Human Kinetics, Inc.

Human Kinetics
ORIGINAL INVESTIGATION

Should We Base Training Prescription on the Force–Velocity Profile? Exploratory Study of Its Between-Day Reliability and Differences Between Methods

Pedro L. Valenzuela, Guillermo Sánchez-Martínez, Elaia Torrontegi, Javier Vázquez-Carrión, Zigor Montalvo, and G. Gregory Haff

Purpose: To analyze the differences in the force–velocity (*F*–*v*) profile assessed under unconstrained (ie, using free weights) and constrained (ie, on a Smith machine) vertical jumps, as well as to determine the between-day reliability. ***Methods:*** A total of 23 trained participants (18 [1] y) performed an incremental load squat jump test (with ~35%, 45%, 60%, and 70% of the subjects' body mass) on 2 different days using free weights and a Smith machine. Nine of these participants repeated the tests on 2 other days for an exploratory analysis of between-day reliability. *F*–*v* variables (ie, maximum theoretical force [F_0], velocity [v_0], and power, and the imbalance between the actual and the theoretically optimal *F*–*v* profile) were computed from jump height. ***Results:*** A poor agreement was observed between the *F*–*v* variables assessed under constrained and unconstrained conditions (intraclass correlation coefficient [ICC] < .50 for all). The height attained during each single jump performed under both constrained and unconstrained conditions showed an acceptable reliability (coefficient of variation < 10%, ICC > .70). The *F*–*v* variables computed under constrained conditions showed an overall good agreement (ICC = .75–.95 for all variables) and no significant differences between days ($P > .05$), but a high variability for v_0, the imbalance between the actual and the theoretically optimal *F*–*v* profile, and maximal theoretical power (coefficient of variation = 17.0%–27.4%). No between-day differences were observed for any *F*–*v* variable assessed under unconstrained conditions ($P > .05$), but all of them presented a low between-day reliability (coefficient of variation > 10% and ICC < .70 for all). ***Conclusions:*** *F*–*v* variables differed meaningfully when obtained from constrained and unconstrained loaded jumps, and most importantly seemed to present a low between-day reliability.

Keywords: muscle properties, assessment, biomechanics, muscle function, strength, power

Optimum levels of lower-limb muscle power constitute a major determinant of performance in a variety of sports—especially those that require the execution of explosive or ballistic movements such as sprinting or jumping.[1] The assessment and improvement of muscle power are therefore relevant.[1] In this regard, the evaluation of lower-limb muscle function through the assessment of the force–velocity (*F*–*v*) profile has recently become very popular.[2] This method consists of modeling force and velocity data collected under 2 or more loaded conditions (eg, loaded jumps or sprints) in order to estimate the theoretical maximal levels of force (F_0, ie, theoretically maximum isometric force), velocity (v_0, ie, theoretically maximum unloaded velocity), and power (P_{max}) that an athlete can produce.[3] Of note, although force plates are considered the "gold standard" method for the assessment of force and velocity, field-based methods (known as Samozino method) have been proposed to estimate the mean values of force and velocity during jumps from 3 simple variables (ie, system mass [eg, athlete's body mass + external load], jump height, and push-off distance).[4] When compared with force plate methods of assessing *F*–*v* variables, these field-based methods have a high concurrent validity.[5]

It has been proposed that although jump height is largely determined by P_{max}, it is also influenced by the individual combination of the underlying force and velocity mechanical outputs.[2,3,6] Thus, the same P_{max} can be attained with different combinations of F_0 and v_0, resulting in different jump performance.[3] In this regard, it has been proposed that there is a theoretically optimal *F*–*v* profile for each individual who would maximize performance for a given P_{max}.[2,6] Recent studies have shown that training that targets the individual *F*–*v* imbalance (F–v_{IMB}, ie, the imbalance between the actual and the theoretically optimal *F*–*v* profile, which might reflect either a force or velocity deficit) results in greater performance gains when compared with a standard training intervention.[7–9] However, while there is some evidence that this method of training is viable, much more research is needed in order to better understand the actual effectiveness of *F*–*v* profile-based training programs when compared with other traditional training interventions.

Although the abovementioned studies support the assessment of the *F*–*v* profile with the Samozino method and particularly the development of a F–v_{IMB} score that can be used to guide training prescription, there is still debate on the optimal method for its determination. Whereas some authors[9–13] have conducted the test under unconstrained conditions (ie, using free weights)—as originally proposed[6], others have combined both unconstrained (for unloaded jumps) and constrained jumps (using a Smith machine for loaded jumps).[5,7,8,14] While both methods have been used, it has yet

Valenzuela is with the Dept of Systems Biology, School of Medicine, University of Alcalá, Madrid, Spain. Valenzuela, Sánchez-Martínez, Torrontegi, Vázquez-Carrión, and Montalvo are with the Dept of Sport and Health, Spanish Agency for Health Protection in Sport (AEPSAD), Madrid, Spain. Haff is with the Human Performance Laboratory, Sport, Exercise, and Physiotherapy, University of Salford, Salford, United Kingdom; and the School of Exercise and Health Sciences, Edith Cowan University, Joondalup, WA, Australia. Valenzuela (pedrol.valenzuela@edu.uah.es) is corresponding author.

AUTHOR PROOF

to be determined whether the data obtained through these 2 assessment methods are interchangeable.

Moreover, although there is evidence that supports the reliability of the F–v profile when it is computed using linear position transducers or force plates,[15–17] to our knowledge no evidence exists examining the between-day reliability of the F–v profile assessed using Samozino method and the reliability of the F–v_{IMB} score, which would be necessary for this method to be used for Q4 training prescription and evaluation. Recent research has reported that even small biomechanical changes (variation of ±10° in the knee angle) can meaningfully influence the F–v variables assessed during loaded squat jumps.[12] In addition, it has recently been reported that although F–v variables obtained during 2 consecutive blocks of jumps present an overall acceptable reliability, the reliability of other important variables such as the F–v slope—from which the F–v_{IMB} is calculated—seems to be poorer (coeffi-
Q5 cient of variation [CV] >10%).[11]

In this context, the aim of the present study was to analyze the differences in F–v variables computed using the Samozino method from loaded jumps performed under unconstrained and constrained conditions, as well as to perform an exploratory analysis to determine the between-day reliability of the F–v profile—and particularly F–v_{IMB}.

Methods

Subjects

A total of 23 Judo athletes aged 17–20 years (mean [SD], age = 18 [1] y; weight = 77 [12] kg, height = 175 [5] cm) who competed at the National level volunteered to participate in this study. Subjects performed 3 to 4 training sessions per week (each lasting 90–120 min) of specific Judo training and had a minimum experience of 2 years performing resistance training (≥2 sessions per week, each lasting 90–120 min), including loaded jump squats. The study took place during a noncompetitive part of the season to avoid weight changes (which are typical in this sport between competitions) or fatigue. All subjects had the procedures explained and provided written informed consent. Parental or guardian consent was required when the participant was under 18 years old. The study was performed in agreement with the Declaration of Helsinki and was approved by the institutional review board (University of Alcalá, Spain). Subjects were instructed to maintain their normal dietary pattern during the study, as well as to refrain from doing intense exercise and from consuming ergogenic aids (eg, creatine, caffeine).

Experimental Design

The present study followed a randomized crossover design and consisted of 2 different experiments. For the magnitude comparison between the F–v variables (ie, F_0, v_0, F–v_{IMB}, P_{max}, and jump height) assessed using free weights (ie, an Olympic barbell) and a Smith machine (Multipower Fitness Line; Peroga, Murcia, Spain), all 23 subjects participated in the experiment. All subjects came to the laboratory on 2 different days, performing an incremental load test in a randomized order in each session. Moreover, 9 of these participants returned to the laboratory on 2 additional occasions, repeating each incremental load test on the Smith machine and free weights in order to evaluate the between-day reliability. All sessions were separated by 48 to 72 hours and were conducted at the same time of the day. Each participant performed all incremental load tests using the same loads across sessions.

Procedures

All procedures were performed in accordance with previously published methods.[7–9] Subjects' body mass and height were measured immediately before each testing session using an automatic scale and a wall-mounted stadiometer (Seca, Barcelona, Spain). The lower limb length was measured using a tape measure (Lufkin W606PM; Apex tool, Sparks, MA) with an accuracy of 0.1 cm. First, with each participant in a supine position with the ankle fully extended, the distance from the iliac crest to the toes was measured. Then, the distance from the iliac crest to the ground was measured, while the participant was in a squatted position (90° of knee flexion). The push-off distance (h_{PO}) was then computed as the difference between these 2 measurements.[3,6] Participants were then familiarized with this squatting position and were instructed to start all squat jumps from this exact position. We also determined the downward displacement of the Olympic barbell—which was placed on the participants' shoulders—between the standing up and the squatting position to ensure that a similar push-off distance (± 1–3 cm approximately) was achieved in all jumps for each participant. For this purpose, a reflective marker was placed on the bar, and its displacement was measured with an infrared optoelectronic system (Velowin, Realtec, Murcia, Spain).[18] Q6

After a standardized 15-minute warm-up (5 min of jogging, 5 min of joint mobility, and 5 min in which participants performed preparatory squat jumps with moderate loads), participants started the incremental load test, performing between 3 and 5 jumps with each load (3 valid jumps were required for each load). Participants performed squat jumps with no added load (0% of the subjects' body mass, performed holding a stick to simulate the Olympic barbell) and with 4 different loads corresponding to approximately 35% (25 [0] kg), 45% (35 [4] kg), 60% (44 [6] kg), and 70% (55 [11] kg) of the subjects' body mass, respectively, in a randomized order. Loaded jumps were performed on a Smith machine or using an Olympic barbell, depending on the assigned condition. Each jump was separated by a 30-second rest and each load by a 5-minute rest. Two researchers ensured that the technique of the jumps was correct during each testing session.

Jump height was calculated from flight time using a photoelectrical contact platform (OptoGait 1.9.9.0; Microgait, Bolzano, Italy). This system has previously been determined to be a valid and reliable tool for the assessment of jump height.[19] The highest jump from the 3 selected valid jumps was used for analysis. F–v variables (ie, F_0, v_0, F–v_{IMB}, P_{max}) were computed for each participant and session based on the subjects' body mass, h_{PO}, and load height as explained elsewhere.[3,6] Briefly, the mean force (F) and velocity (v) were computed for each jump, and F–v curves were extrapolated to obtain F_0 and v_0, which correspond to the intercepts of the F–v curve with the force and velocity axis, respectively. P_{max} was determined as the product of F_0 and v_0 divided by 4.[3,6] We also computed the F–v_{IMB} (as a percentage) by comparing the slope of the actual F–v profile with the slope of the theoretically optimal F–v profile as proposed by Samozino et al.[6] We checked the linearity of the F–v relationship in the spectrum of data available (55.3%–90.6% F_0, 0.65–1.38 $m \cdot s^{-1}$) by considering the correlation coefficients (R^2 = .97 [.03] and .96 [.04] for the tests performed with the Smith machine and with free weights, respectively).

Statistical Analysis

Following the calculations performed by previous studies on the F–v profile, a sample size of ~12 was considered sufficient to detect differences in F–v variables with an alpha level <.05 and a power

AUTHOR PROOF

Table 1 Comparison of Force–Velocity Variables Computed During an Incremental Squat Jump Test Under Constrained (ie, on a Smith Machine) and Unconstrained Conditions (ie, Using Free Weights)

Variable	Smith	Free weights	*P* value	ES	CV, %	SEM (90% CI)	ICC (90% CI)
F_0, N·kg^{-1}	28.6 (3.4)	30.7 (2.8)	.008	0.64	8.0	2.36 (1.90 to 3.15)	.45 (.13 to .69)
V_0, m·s^{-1}	4.40 (1.00)	3.89 (1.24)	.104	0.36	24.9	1.02 (0.82 to 1.36)	.20 (–.16 to .51)
F–v_{IMB}, %	57.2 (14.9)	47.1 (17.2)	.025	0.50	27.4	14.30 (11.52 to 19.10)	.22 (–.13 to .52)
P_{max}, W·kg^{-1}	31.0 (5.7)	29.5 (8.3)	.308	0.21	17.0	5.12 (4.12 to 6.83)	.50 (.19 to .72)
H1, cm	19.4 (3.4)	20.6 (3.6)	<.001	0.98	4.5	0.90 (0.73 to 1.21)	.94 (.88 to .97)
H2, cm	16.3 (2.7)	17.5 (3.7)	.022	0.52	9.9	1.67 (1.35 to 2.23)	.75 (.55 to .87)
H3, cm	13.5 (2.3)	14.6 (2.0)	<.001	0.94	6.0	0.84 (0.68 to 1.12)	.86 (.73 to .939)
H4, cm	10.9 (1.7)	11.7 (1.2)	.005	0.61	8.1	0.92 (0.74 to 1.23)	.62 (.36 to .80)

Abbreviations: CI, confidence interval; CV, coefficient of variation; ES, effect size (Hedges *g*); F_0, maximal theoretical force; F–v_{IMB}, force–velocity imbalance; H, jump height; ICC, intraclass correlation coefficient; P_{max}, maximal theoretical power; SEM, standard error of measurement; V_0, maximal theoretical velocity. Note: H1, H2, H3 and H4 correspond to the height attained during jumps performed with 35%, 45%, 60%, and 70% of participants' body mass. Data are presented as mean (SD).

>0.80.[16] Given that some of our analyses were performed with a lower sample size (ie, n = 9 for reliability analyses), our results should be considered exploratory.

Data are presented as mean (SD). Differences between conditions and between days were assessed using Student paired *t* tests. The magnitude of these differences was assessed using effect sizes (ES, Hedge *g*), which were computed using G*Power (version 3.1.9.2; Heinrich Heine Universität Düsseldorf, Düsseldorf, Germany) and considered trivial (ES < 0.20), small (ES < 0.60), moderate (ES < 1.20), large (ES < 2.00), or very large (ES < 4.00).[20] Consecutive pairwise intraclass correlation coefficients (ICCs) and standard errors of measurement (expressed along with upper and lower 90% confidence intervals) were calculated with a spreadsheet for the analysis of the agreement between measures.[21] CVs (computed as the ratio between the standard errors of measurement and the mean of the measures) were also computed. Acceptable reliability was determined as a CV < 10% and an ICC > 0.70.[22] Statistical analyses were conducted using a statistical software package (SPSS, version 23.0; IBM Corp, Armonk, NY) setting the significance level at $\alpha = .05$.

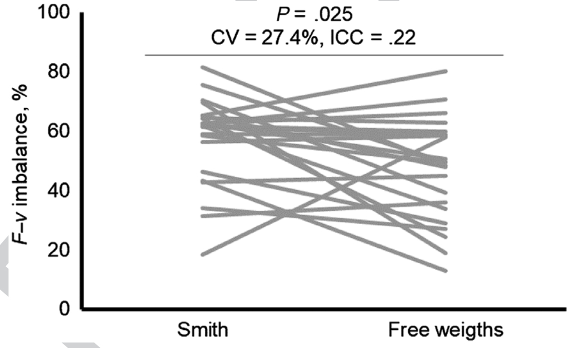

Figure 1 — Individual differences on the *F*–*v* imbalance assessed under constrained (ie, on a Smith machine) and unconstrained conditions (ie, using free weights). The *P* value corresponds to the comparison between conditions. CV indicates coefficient of variation; *F*–*v*, force–velocity; ICC, intraclass correlation coefficient.

Q7

Results

Magnitude Comparison

The variables obtained on the Smith machine and using free weights are shown in Table 1. Significantly lower jump heights (ES = 0.52–0.98) were found for the Smith machine compared with the free-weight condition. The values of jump height attained on the Smith machine and using free weights were reliable (CV < 10%, ICC > .70), except for the heaviest load (ICC = .62 for 75 kg).

On the other hand, the *F*–*v* variables computed from these jumps showed a poor agreement (ICC < .50 for all), poor reliability (CV ranging from 17.0% to 24.9% for v_0, F–v_{IMB} and P_{max}), or significant differences between conditions ($P < .05$ for F_0 and F–v_{IMB}). Individual data for the F–v_{IMB} computed on a Smith machine and using free weights are shown in Figure 1.

Reliability Analysis

Reliability analyses are shown in Table 2. The height attained during the jumps performed on both the Smith machine (CV = 4.4%–7.8%, ICC = .85–.96) and using free weights (CV = 3.8%–7.8%, ICC = .69–.96) showed an acceptable reliability and nonsignificant differences between days.

The *F*–*v* variables computed in the Smith machine had an overall good agreement and no significant differences between days (ICC = .75–.95), but a high CV (>10%) for v_0, F–v_{IMB}, and P_{max}. In turn, all computed *F*–*v* variables (ie, F_0, v_0, F–v_{IMB}, and P_{max}) from the tests with free weights showed a low reliability (CV > 10% and ICC < .70). Individual data for the F–v_{IMB} computed during each testing session on a Smith machine and using free weights are shown in Figure 2.

Discussion

The main findings of the present exploratory study are that (1) the jump heights attained during loaded jumps (from 0% to ~70% of the athletes' body mass) performed under constrained and unconstrained conditions are reliable (CV < 10%, ICC > 0.70), but their magnitude significantly differ between conditions; (2) the *F*–*v* variables estimated from these jump heights (ie, F_0, V_0, F–v_{IMB}, and P_{max}) using Samozino method present significant differences and/or a low ICC (<.50) between constrained and unconstrained conditions, and thus the computed values are not interchangeable; and (3) the *F*–*v* variables and particularly the F–v_{IMB} estimated from loaded jumps performed under constrained and especially during unconstrained conditions present a high between-day variability (CV > 10%).

AUTHOR PROOF

Table 2 Between-Day Reliability of Force–Velocity Variables Computed During an Incremental Squat Jump Test Under Constrained (ie, on a Smith Machine) and Unconstrained Conditions (ie, Using Free Weights)

Q8

	Variable	Day 1	Day 2	*P* value	ES	CV, %	SEM (90% CI)	ICC (90% CI)
Smith	F_0, N·kg^{-1}	28.7 (4.0)	28.1 (3.4)	.205	0.43	3.4	0.97 (0.70 to 1.66)	.95 (.84 to .99)
	V_0, m·s^{-1}	4.26 (0.89)	4.79 (1.24)	.090	0.66	12.6	0.57 (0.41 to 0.98)	.77 (.38 to .93)
	F–v_{IMB}, %	56.6 (16.6)	61.7 (14.6)	.166	0.51	12.1	7.14 (5.13 to 12.21)	.84 (.54 to .95)
	P_{max}, W·kg^{-1}	30.2 (4.9)	33.2 (7.2)	.105	0.62	11.0	3.47 (2.49 to 5.93)	.75 (.33 to .92)
	H0, cm	30.9 (4.8)	30.8 (6.0)	.925	0.03	7.8	2.42 (1.74 to 4.14)	.85 (.58 to .95)
	H1, cm	18.7 (3.7)	19.3 (3.6)	.235	0.45	4.9	0.94 (0.67 to 1.60)	.95 (.85 to .99)
	H2, cm	16.1 (2.8)	16.3 (2.9)	.608	0.20	4.4	0.71 (0.51 to 1.21)	.96 (.86 to .99)
	H3, cm	13.2 (2.2)	13.6 (2.4)	.485	0.34	6.3	0.84 (0.60 to 1.43)	.90 (.71 to .97)
	H4, cm	11.1 (2.1)	10.7 (1.5)	.259	0.41	6.6	0.72 (0.52 to 1.23)	.89 (.65 to .97)
Free weights	F_0, N·kg^{-1}	29.9 (3.6)	31.5 (2.4)	.305	0.38	9.9	3.03 (2.18 to 5.19)	.04 (−.52 to .57)
	V_0, m·s^{-1}	4.05 (0.89)	3.83 (1.77)	.740	0.11	34.5	1.36 (0.98 to 2.32)	.07 (−.50 to .60)
	F–v_{IMB}, %	52.6 (16.7)	43.2 (19.5)	.354	0.42	42.1	20.2 (14.5 to 34.5)	−.27 (−0.71 to .33)
	P_{max}, W·kg^{-1}	29.9 (5.8)	29.7 (12.1)	.944	0.02	30.0	7.75 (5.57 to 13.26)	.38 (−.21 to .77)
	H0, cm	30.9 (4.8)	30.8 (6.0)	.925	0.03	7.8	2.42 (1.74 to 4.14)	.85 (.58 to .95)
	H1, cm	20.3 (4.1)	20.2 (4.0)	.763	0.08	4.4	0.90 (0.65 to 1.55)	.96 (.88 to .99)
	H2, cm	16.9 (2.8)	17.3 (2.5)	.108	0.40	3.8	0.65 (0.47 to 1.11)	.95 (.85 to .99)
	H3, cm	14.3 (2.1)	14.8 (1.6)	.224	0.48	5.0	0.73 (0.53 to 1.25)	.89 (.67 to .97)
	H4, cm	11.6 (1.3)	12.0 (1.4)	.385	0.34	6.9	0.82 (0.59 to 1.40)	.69 (.22 to .90)

Abbreviations: CI, confidence interval; CV, coefficient of variation; ES, effect size (Hedges *g*); F_0, maximal theoretical force; F–v_{IMB}, force–velocity imbalance; H, jump height; ICC, intraclass correlation coefficient; P_{max}, maximal theoretical power; SEM, standard error of measurement; V_0, maximal theoretical velocity. Note: H0, H1, H2, H3 and H4 correspond to the height attained during jumps performed with 0%, 35%, 45%, 60%, and 70% of participants' body mass. Data are presented as mean (SD).

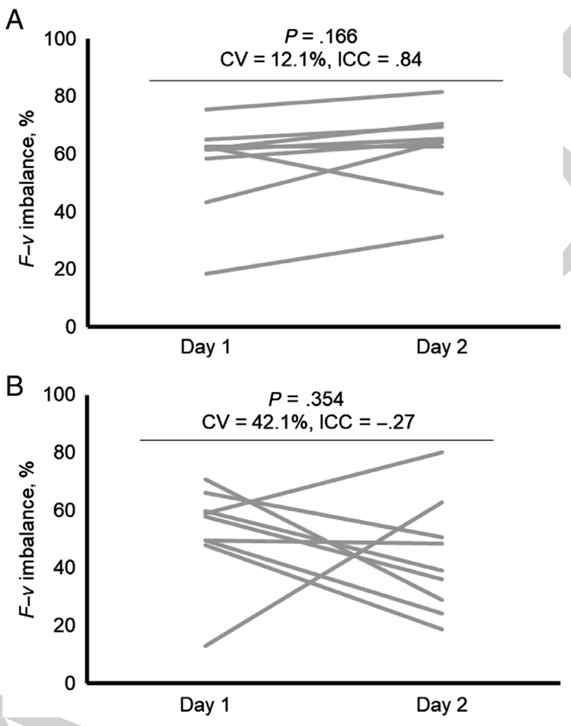

Figure 2 — Individual between-day variation of the *F*–*v* imbalance assessed under constrained (ie, on a Smith machine, A) or unconstrained conditions (ie, using free weights, B). The *P* value corresponds to the comparison between days. CV indicates coefficient of variation; *F*–*v*, force–velocity; ICC, intraclass correlation coefficient.

In line with our results, previous evidence supports the reliability of jump height under both constrained and unconstrained conditions,[23,24] although a higher variability seems to be present with heaviest loads when using free weights (eg, CV = 19% and ICC = .74 for 75 kg).[23] There is inconsistent evidence related to jump height between conditions, with some authors finding no differences[24] and others reporting greater jump heights during testing sessions performed with a Smith machine, particularly with heavier loads (difference of 9% with 75 kg).[23] However, in the present study we observed greater jump heights under unconstrained conditions, which could be partially explained by the influence of friction forces associated with the Smith machine.

Some debate also exists on the best method (ie, Smith vs free weights) for the assessment of the *F*–*v* profile. In their seminal study, Samozino et al[6] computed the F–v_{IMB} based on the jump height attained during a series of loaded tests performed with an Olympic barbell. Numerous studies have followed this or a similar procedure, performing both unloaded and loaded jumps using free weights.[9–13] In turn, several recent studies have estimated the F–v_{IMB} with loaded jumps performed under constrained conditions.[5,7,8,14] Moreover, some of these studies prescribed training interventions based on the computed F–v_{IMB}, with some using free weights[9] and others using a Smith machine.[7,8] In this regard, our study presents data that F–v_{IMB} values—as well as the rest of *F*–*v* variables—present a low agreement between measurements made with free weights or a Smith machine. This lack of agreement could result in an individual being prescribed the wrong training (eg, focusing on a velocity vs focusing on a force deficit) depending on the assessment method and whether that method corresponds to the training methods. Future research should confirm whether a mathematical correction can be made attending to variables such as friction forces—which are usually overlooked but can

AUTHOR PROOF

be assessed using a simple freefall test, as done elsewhere for the estimation of mean force and velocity during the bench press[25]—to normalize the measures obtained under constrained and unconstrained conditions.

Q9 Finally, one of the major findings of our study was the low reliability observed for the *F–v* variables computed from loaded jumps performed under constrained and particularly unconstrained conditions, despite the acceptable reliability of each individual jump. Previous evidence supports an overall acceptable reliability of the *F–v* profile or load–velocity profile of squat and jump exercises computed using linear position transducers[15] and force plates.[16,17] However, to our knowledge no previous study had examined the between-day reliability of the important *F–v* variables (F_0, v_0, P_{max}, and F–v_{IMB}) computed using Samozino method. Samozino et al[4] and Giroux et al[5] observed that the mean *F–v* variables computed for each individual jump (*F*, *v*, and *P*, but not F_0, v_0, P_{max}, or F–v_{IMB}) using Samozino method presented a good between-day reliability. Moreover, Janicijevic et al[11] recently observed that the *F–v* variables obtained with Samozino method during 2 consecutive blocks of jumps (computing *F–v* variables based on 1 unloaded jump and 2 loaded jumps performed using free weights) presented an overall acceptable reliability. However, whereas the reliability was high for variables such as F_0 or P_{max} (CV < 5% and ICC > .90 when using both a self-preferred knee angle and a fixed knee angle of 90°), the reliability for the slope of the *F–v* profile was poorer (CV > 10% and ICC < .80).[11] Moreover, the same research group recently reported that the *F–v* variables assessed during squat jumps (again, based on 1 unloaded jump and 2 loaded jumps performed using free weights) differ meaningfully when obtained with a knee angle of 80°, 90°, or 100°, with the *F–v* slope being indeed poorly correlated between conditions (r = .178–.645).[12] These results highlight the importance of h_{PO} on the *F–v* profile, as variations in the h_{PO} can result in meaningful differences in the computed values of force and velocity.[6] As proposed by Samozino et al[26] "for a given individual, a change in h_{PO} (induced for instance by a change in starting position) may lead to variations in F0 and v0 due to the effects of both muscles force-length relationships and changes in joint moment arms during extension." In the present study, we tried to keep h_{PO} steady across trials, but our results suggest that even minor changes might result in a low reliability of *F–v* measures. The accurate monitoring of h_{PO} across tests is therefore essential to ensure the optimal reliability of the *F–v* profile.

Apart from the potential influence of h_{PO} on the reliability of the *F–v* profile, the inner subjects' individual physiological characteristics should not be disregarded. The *F–v* profile depends on the proper fit of the *F–v* data obtained for each individual subject to a linear regression, which allows the estimation of F_0 and v_0. However, considerable debate exists regarding the actual linearity of *F–v* data, particularly at very low force values, which could not be measured in the present study.[27,28] It is important to note that factors that impact the linearity of the measure might result in an incorrect estimation of V_0 and F–v_{IMB}. In addition, some *F–v* data might deviate from this linearity due to a lack of maximal effort by the subject during the prescribed jumps. Future research should confirm if the *F–v* data estimated by Samozino method is actually linear, and address whether setting objective thresholds for determining the validity of the obtained *F–v* data from each jump, as shown in a recent study for the *F–v* profile assessed during the leg press exercise with a linear position transducer,[29] could improve the reliability of this method.

Q10

Practical Applications

The present findings suggest that *F–v* variables assessed using Samozino method differ meaningfully when obtained using constrained and unconstrained jumps, which can result in an individual presenting a completely different F–v_{IMB} (ie, force vs velocity deficit) depending on the assessment method. Most importantly, our results show that—contrary to the height attained in each single jump—the estimated *F–v* variables and particularly the F–v_{IMB} present a poor reliability between days when obtained under both constrained and especially unconstrained conditions. These findings raise concerns on the suitability of these markers—at least in the present population and when obtained following the procedures used here—for the accurate assessment of muscle function or for the guidance of training prescription. Indeed, the observed standard errors of measurement for F–v_{IMB} (7.1% and 20.2% for constrained and unconstrained conditions) are close to or even higher than the threshold (±10%) set by other authors to determine whether a *F–v* profile is balanced or not, and could therefore result in a subject being prescribed a diverse training prescription depending on the daily variation in the *F–v* profile.[7,8] Further research is therefore needed to improve the reliability of this procedure, as well as to confirm whether friction forces should be considered when testing the *F–v* profile under constrained conditions. More studies are also warranted to compare the effectiveness of *F–v* profile-based training programs and traditional training interventions (eg, plyometrics, mixed training programs, etc). Indeed, despite being outside the scope of this work, it must be noted that recent evidence suggests that an individualized training intervention based on the "horizontal" *F–v* profile (ie, computed from sprints instead of jumps) is not more effective than a standard one for the improvement of sport performance.[30] Moreover, a recent study reported that, although an individualized training intervention prescribed attending to the "vertical" *F–v* profile led to greater improvements on the F–v_{IMB} than a nonindividualized training program, it provided no consistent additional benefits (ie, no significant time by group interaction effect) on power, sprint, or strength performance.[13]

Finally, some limitations must be acknowledged, notably the small sample size used—particularly for reliability analyses—and not having replicated our procedures using a gold standard method such as a force plate. Moreover, the characteristics of the included participants who were young trained judokas and do not usually jump as part of their sport (despite being familiarized with the loaded jump squat) might also potentially confound the results. In this regard, it must be noted that the *F–v* profile assessed through Samozino method has been used for the assessment of lower-limb mechanical properties in a great variety of sports (eg, basketball, rugby, volleyball, judo, karate, weightlifting, tennis).[31] The level of familiarization with the test procedures (ie, loaded squat jumps) needed for an optimal reliability of the *F–v* profile should be investigated, and research is also warranted to confirm whether this strategy is appropriate—based on its validity, reliability, and sensitivity—for the assessment of lower-limb performance in sports in which vertical jumps are not a main component.

Conclusions

Our exploratory analyses show that *F–v* variables (ie, F_0, v_0, F–v_{IMB}, and P_{max}) estimated using Samozino method might differ meaningfully when obtained from either constrained or unconstrained loaded jumps. Moreover, although the height attained on each single jump seems to present an acceptable reliability on both

AUTHOR PROOF

constrained and unconstrained conditions, the abovementioned F–v variables and particularly the F–v_{IMB} might present a low between-day reliability, particularly when obtained from unconstrained loaded jumps.

Acknowledgments

The authors sincerely appreciate the help of Dr Alfonso Díaz López de Durana (Politechnic University of Madrid) for his help with the recruit-
Q11 ment of study participants. The work of P.L.V. is supported by the University of Alcalá (FPI2016). The authors declare no conflicts of interest.

References

1. Cormie P, McGuigan MR, Newton RU. Developing maximal neuromuscular power. *Sports Med*. 2011;41(2):125–146. doi:10.2165/11538500-000000000-00000
2. Morin JB, Samozino P. Interpreting power-force-velocity profiles for individualized and specific training. *Int J Sports Physiol Perform*. 2016;11(2):267–272. PubMed ID: 26694658 doi:10.1123/ijspp.2015-0638
3. Samozino P, Rejc E, Di Prampero PE, Belli A, Morin JB. Optimal force-velocity profile in ballistic movements-altius: citius or fortius? *Med Sci Sports Exerc*. 2012;44(2):313–322. PubMed ID: 21775909 doi:10.1249/mss.0b013e31822d757a
4. Samozino P, Morin JB, Hintzy F, Belli A. A simple method for measuring force, velocity and power output during squat jump. *J Biomech*. 2008;41(14):2940–2945. PubMed ID: 18789803 doi:10.1016/j.jbiomech.2008.07.028
5. Giroux C, Rabita G, Chollet D, Guilhem G. What is the best method for assessing lower limb force-velocity relationship? *Int J Sports Med*. 2014;36(2):143–149. PubMed ID: 25259590 doi:10.1055/s-0034-1385886
6. Samozino P, Edouard P, Sangnier S, Brughelli M, Gimenez P, Morin JB. Force-velocity profile: Imbalance determination and effect on lower limb ballistic performance. *Int J Sports Med*. 2014;35(6):505–510. PubMed ID: 24227123 doi:10.1055/s-0033-1354382
7. Jiménez-Reyes P, Samozino P, Brughelli M, Morin JB. Effectiveness of an individualized training based on force-velocity profiling during jumping. *Front Physiol*. 2017;7:1–13. doi:10.3389/fphys.2016.00677
8. Jiménez-Reyes P, Samozino P, Morin JB. Optimized training for jumping performance using the force-velocity imbalance: individual adaptation kinetics. *PLoS One*. 2019;14(5):1–20. doi:10.1371/journal.pone.0216681
9. Escobar Álvarez JA, Fuentes García JP, Da Conceição FA, Jiménez-Reyes P. Individualized training based on force–velocity profiling during jumping in ballet dancers [published online ahead of print
Q12 November 11, 2019]. *Int J Sports Physiol Perform*. PubMed ID: 31715582 doi:10.1123/ijspp.2019-0492
10. Marcote-Pequeño R, García-Ramos A, Cuadrado-Peñafiel V, González-Hernández JM, Gómez MÁ, Jiménez-Reyes P. Association between the force–velocity profile and performance variables obtained in jumping and sprinting in elite female soccer players. *Int J Sports Physiol Perform*. 2019;14(2):209–215. PubMed ID: 30040003 doi:10.1123/ijspp.2018-0233
11. Janicijevic D, Knezevic OM, Mirkov DM, et al. Assessment of the force-velocity relationship during vertical jumps: influence of the starting position, analysis procedures and number of loads. *Eur J*
Q13 *Sport Sci*. 2020;20:614–623. doi:10.1080/17461391.2019.1645886
12. Janicijevic D, Knezevic O, Mirkov D, et al. The force-velocity relationship obtained during the squat jump exercise is meaningfully influenced by the initial knee angle. *Sports Biomech*. 2020. Q14
13. Zabaloy S, Pareja-Blanco F, Giráldez JC, Rasmussen JI, González JG. Effects of individualised training programmes based on the force-velocity imbalance on physical performance in rugby players. *Isokinet Exerc Sci*. 2020;28(2):181–190. doi:10.3233/ies-192196
14. García-Ramos A, Pérez-Castilla A, Morales-Artacho AJ, et al. Force-velocity relationship in the countermovement jump exercise assessed by different measurement methods. *J Hum Kinet*. 2019;67(1):37–47. doi:10.2478/hukin-2018-0085
15. Iglesias-Soler E, Mayo X, Rial-Vázquez J, et al. Reliability of force-velocity parameters obtained from linear and curvilinear regressions for the bench press and squat exercises. *J Sports Sci*. 2019;37(22):2596–2603. doi:10.1080/02640414.2019.1648993
16. Cuk I, Markovic M, Nadeljkovic A, Ugarkovic D, Kukolj M, Jaric S. Force-velocity relationship of leg extensors obtained from loaded and unloaded vertical jumps Ivan. *Eur J Appl Physiol*. 2014;114(8):1703–1714. PubMed ID: 24819449 doi:10.1038/jid.2014.371
17. Feeney D, Stanhope SJ, Kaminski TW, Machi A, Jaric S. Loaded vertical jumping: force-velocity relationship, work, and power. *J Appl Biomech*. 2016;32(2):120–127. PubMed ID: 26398963 doi:10.1123/jab.2015-0136
18. Laza-Cagigas R, Goss-Sampson M, Larumbe-Zabala E, Termkolli L, Naclerio F. Validity and reliability of a novel optoelectronic device to measure movement velocity, force and power during the back squat exercise. *J Sports Sci*. 2018;37(7):795–802. PubMed ID: 30306839 doi:10.1080/02640414.2018.1527673
19. Glatthorn JF, Gouge S, Nussbaumer S, Stauffacher S, Impellizzeri FM, Maffiuletti NA. Validity and reliability of optojump photoelectric cells for estimating vertical jump height. *J Strength Cond Res*. 2011;25(2):556–560. PubMed ID: 20647944 doi:10.1519/jsc.0b013e3181ccb18d
20. Hopkins W, Marshall SW, Batterham AM, Hanin J. Progressive statistics for studies in sports medicine and exercise science. *Med Sci Sports Exerc*. 2009;41(1):3–12. PubMed ID: 19092709 doi:10.1249/mss.0b013e31818cb278
21. Hopkins W. Spreadsheets for analysis of validity and reliability. *Sportscience*. 2015;19:36–42. sportsci.org/2015/ValidRely.htm.
22. Cormack SJ, Newton RU, McGulgan MR, Doyle TLA. Reliability of measures obtained during single and repeated countermovement jumps. *Int J Sports Physiol Perform*. 2008;3(2):131–144. PubMed ID: 19208922 doi:10.1123/ijspp.3.2.131
23. Pérez-Castilla A, McMahon JJ, Comfort P, García-Ramos A. Assessment of loaded squat jump height with a free-weight barbell and Smith machine: comparison of the take-off velocity and flight time procedures. *J Strength Cond Res*. 2020;34(3):671–677. PubMed ID: 28777251 doi:10.1519/jsc.0000000000002166
24. García-Ramos A, Jaric S, Pérez-Castilla A, Padial P, Feriche B. Reliability and magnitude of mechanical variables assessed from unconstrained and constrained loaded countermovement jumps. *Sports Biomech*. 2017;16(4):514–526. doi:10.1080/14763141.2016.1246598
25. Rahmani A, Samozino P, Morin J-B, Morel B. A simple method for assessing upper limb force-velocity profile in bench press. *Int J Sports Physiol Perform*. 2017;13(2):200–207. doi:10.1123/ijspp.2016-0814
26. Samozino P, Morin JB, Hintzy F, Belli A. Jumping ability: a theoretical integrative approach. *J Theor Biol*. 2010;264(1):11–18. PubMed ID: doi:10.1016/j.jtbi.2010.01.021 20109471
27. Alcazar J, Pareja-Blanco F, Rodriguez-Lopez C, et al. Comparison of linear, hyperbolic and double-hyperbolic models to assess the force-velocity relationship in multi-joint exercises [published online ahead of print May 4, 2020]. *Eur J Sport Sci*. PubMed ID: 32255757 doi:10. Q15
1080/17461391.2020.1753816

AUTHOR PROOF

28. Farris DJ, Lichtwark GA, Brown NAT, Cresswell AG. Deconstructing the power resistance relationship for squats: a joint-level analysis. *Scand J Med Sci Sports*. 2016;26(7):774–781. PubMed ID: 26103786 doi:10.1111/sms.12508
29. Alcazar J, Rodriguez-Lopez C, Ara I, et al. The force-velocity relationship in older people: reliability and validity of a systematic procedure. *Int J Sports Med*. 2017;38(14):1097–1104. PubMed ID: 29126339 doi:10.1055/s-0043-119880
30. Rakovic E, Paulsen G, Helland C, Eriksrud O, Haugen T. The effect of individualised sprint training in elite female team sport athletes: a pilot study. *J Sports Sci*. 2018;36(24):2802–2808. PubMed ID: 29741443 doi:10.1080/02640414.2018.1474536
31. Jiménez-Reyes P, Samozino P, García-Ramos A, Cuadrado-Peñafiel V, Brughelli M, Morin JB. Relationship between vertical and horizontal force-velocity-power profiles in various sports and levels of practice. *PeerJ*. 2018;2018(11):1–18. doi:10.7717/peerj.5937

AUTHOR PROOF
