# Supplementary material for: Force-velocity profiling in athletes: Reliability and agreement across methods
Source: PLoS One. 2021 Feb 1;16(2):e0245791. doi: 10.1371/journal.pone.0245791 (PMC7850492; doi:10.1371/journal.pone.0245791)
Supplement: Attachment — Submitted filename: Response to Reviewers.pdf [file pone.0245791.s003.pdf]

We would like to thank the reviewers for a thorough and excessive job of reviewing this manuscript. The inputs and suggestions from the referees have been taken under consideration and detailed responses to reviewers are given below. Please note that the reviewer comments are copied in below with **bold text**, and the changes made in the revised version of the manuscript are in red and explained directly below.

Reviewer #1: The present study assessed the between-day reliability of the f-v parameters computed from different exercises (CMJ, SJ and leg press) and using different methods (force plate, encoder, flight time), as well as the differences in the magnitude of the f-v parameters assessed with these methods. Additionally, the authors examined the association of these parameters with some performance measures.

I think the topic is relevant, particularly given the growing popularity of these methods for guiding training prescription and for performance assessment. Moreover, the results are quite novel and could have practical implications. My main concern is that the manuscript (including the text, figures and tables) is a bit difficult to follow in some parts, and it could maybe be simplified to enhance readability. Moreover, some typos should be corrected. Specific comments:

Thank you for your comments and thorough job of reviewing the manuscript. We have answered all your comments below and show changes in the manuscript fil with the "track changes" function.

Abstract

- Line 24. The athletes 'were' tested…?

Thank you, the sentence is modified accordingly "The athletes were tested twice before and twice after a 2-6-month period of regular training and sport participation" (page 1, line 31)

- Line 41. I suggest including in the last sentence of the abstract that coaches should also be aware of the low reliability of f-v parameters (particularly v0 and Sfv) assessed during jumping.

Thank you, we have modified the last sentence accordingly: " Coaches and researchers should be aware of the poor reliability of the FV-variables obtained from vertical jumping as well as the differences across measurement methods."

(page 1, line 48)

- There is no comment on the association between fv parameters and performance measures. In my opinion, and just as a suggestion that the authors are free to obviate, the manuscript is strong enough when presenting reliability and magnitude comparison methods, and adding the association between fv parameters and performance measures complicates the manuscript. This last section, which is presented as a secondary aim of the study, could be presented even in a separate paper, but this is just in my humble opinion.

Thank you for your comment, we have removed the association between fv parameters and performance measures from the manuscript as recommended.

Introduction

- It would be nice to cite this review, which gives a nice overview of the applications of VBRT (and of the assessment of the fv profile): https://journals.lww.com/nsca-scj/Abstract/9000/Velocity_Based_Training__From_Theory_to.99257.aspx

Thank you, we have now cited the review and added this at page 1, line 53, after the sentence "Within strength and power training, force-velocity (FV) profiling has received increasing attention as means to monitor training adaptations (1-3) and to serve as a basis for individual training prescriptions for athletes (3-6)"

- Line 50. It would be nice to mention that there is some controversy regarding the linearity of the f-v relationship, at least at very low force values (PubMed ID: 32255757, PubMed ID: 26103786).

Thank you for mentioning this. We are aware of the works of Alcazar and are planning some more elaborate experiments regarding this controversy. We highlighted these issues in earlier versions of the manuscript, but it was a bit unclear and long. We have now added this point to the manuscript again, at page 2 line 65:

"The reader should, however, be aware that controversy exist about the linearity of FV-relationships obtained from multi-joint movements (8)."

- Line 57. Can the authors include a reference for this equation? Maybe one of the studies/reviews of Morin and Samozino would be nice.

Thank you, we have added the reference "Samozino P. A simple method for measuring lower limb force, velocity and power capabilities during jumping. Biomechanics of Training and Testing: Springer; 2018. p. 65-96" page 2, line 64

- Line 60. Please, specify how these f-v parameters were computed. From jump height? From force platforms? linear encoders? Has the between-day reliability of the f-v profile estimated from jump height been proven?

Thank you, we have modified and added to the following sentence:

"However, although several studies have evaluated the within-session reliability of FV-parameters (11-19), limited attention has been directed towards the between-session reliability of the FV-parameters in athletes. Additionally, only encoders and the flight time calculation method have been used for measurements of between-session reliability of the FV-parameters (12, 13, 19). Hence, reliability of other commonly used methods such as force plates and leg press devices are unknown (11-18)"

page 2, line 72

- Line 65. Please, specify which measurement methods.

Thank you, we have added to the following sentence:

Giroux et al. (17) have previously investigated the reliability and agreement among three measurement methods (accelerometry, linear position transducer and flight time calculation method) during vertical jumps. However, they reported only average values of force, velocity and power for each jump, and not the extrapolated FV-parameters (F_0, V_0, P_{max} and S_{FV}) that are increasingly used for individual training prescriptions (3-5, 20)

page 2, line 80

- Line 69. Please, specify the method of measurement. Jump height? Force platform?

Thank you, we have added to the following sentence:

García-Ramos et al. (19) have investigated the agreement across methods (force platform, linear position transducer and flight time calculation method) for CMJ, but not SJ.

page 3, line 81

- Line 72. A recent study by Valenzuela et al (accepted in IJSPP, but still in press) reported a

low between-day reliability for f-v parameters computed from jump height during vertical jumps. Please, see attached document. It would be nice to briefly mention this article in the introduction or discussion section.

Thank you for suggesting this interesting paper. We have added this reference to the introduction:

"Additionally, only encoders and the flight time calculation method have been used for measurements of between-session reliability of the FV-parameters (12, 13, 19). Hence, reliability of other commonly used methods such as force plates and leg press devices are unknown (11-18)"

page 2, line 72

And discussion:

"The present results are in accordance with previous research in other populations showing mostly acceptable reliability for F_0 and P_{max} (CV<10%) and poor reliability for V_0 and S_{FV} (CV >10%) during vertical jumping (12, 19, 25, 42, 43)."

page 14, line 346

"The influence of standardisation is also supported by the findings of Pedro L et al. (19) which showed superior reliability of the FV-parameters obtained using a smith machine compared to free weights."

page 15, line 367

- Line 74. Should it be "there exist numerous methods..."?

Thank you, we have added to the following sentence:

"A novel aspect of FV-profiling during vertical jumping is the possibility to obtain the extrapolated variable V_0 and the calculated S_{FV}, as there exists numerous methods for assessing maximal force and maximal power (24)"

page 3, line 85

- Line 87. "...as well as from the leg press exercise".

Thank you, we have added to the following sentence:

"It is therefore of great interest to investigate the reliability of the extrapolated FV-variables from commonly used vertical jumping exercises as well as from the leg press exercise."

page 3, line 98

- Line 88. Previous research "has" investigated...

Thank you for your comment, we have removed the association between fv parameters and performance measures from the manuscript as recommended.

Methods

- Line 134. Which was their experience with the testing procedures?

Thank you, we have added the following sentence:

“The athletes in the main sample were familiar with the testing procedures, whereas the subjects in the mixed sample had various experience prior to the study. “

page 4, line 123

- Line 167. It would be nice to include a new paragraph with each test, as done for SJ and CMJ.

Thank you, the paragraphs are changed accordingly.

- How much time was left between tests?

The time between tests are stated at page 6, line 158 “The different tests were separated by 5-10 min to ensure proper recovery”

- Line 202. It would be nice to start a new paragraph with each measurement method (as done for the Keiser leg press)

Thank you, we have added extra paragraphs.

Results

- Line 259. coma not needed before ‘ranging’.

Thank you, comma removed.

- Line 269. 1.2 cm for both SJ and CMJ?

Yes, the sentence is modified accordingly: The typical error for both SJ and CMJ jump height were 1.2 cm, corresponding to a coefficient of variation of 6.8%

page 11, line 278

- There is no mention to the SWC in the results section. Were differences between days or between methods greater than the SWC?

Correct, the SWC was only presented in figure 3, where readers can compare SWC% with the CV%, to assess whether between day variability is greater than CV%. Similarly, the readers can compare the figure to the systematic bias and random error across methods presented in table 5. Additionally, we refer to the SWC figure at page 18, line 423.

“Additionally, the usefulness of a test is determined by the ability to detect changes in performance, more specifically by comparing the typical error (CV%) with SWC (Helland et al 2020 PeerJ). Indeed, the FV-variables obtained from the leg press apparatus showed superior signal-to-noise ratio compared to the other measurement methods in this study (Figure 3).”

- Line 291. The results on the association between fv parameters and performance measures should be explained in greater detail in the text (if the authors want to keep these analyses). On which tests (CMJ, SJ, leg press) was an association found? With which measurement method? (encoder, force plate, flight time).

These analyses are now removed as suggested above.

Discussion

In my opinion the discussion section could be shortened and simplified. The authors could try to avoid repeating concepts.

Thank you, we agree. The discussion section is reduced from 3031 words to approximately 2300 words.

- Line 298. 'were' unreliable? The authors are mentioning two variables.

Thank you, "were" is added instead of "was"

- Line 332 and Line 423. Small differences in starting position during jumping (impulse distance) can have meaningful effects on the fv variables (PMID: 32223526).

Thank you, we have added these references to the context where this is being discussed:

"superior standardization in terms of push-off distance and fixed body position compared to the free weight conditions during CMJ and SJ (17, 18, 45, 46)"

page 14, line 322

"Additionally, the assumption of constant acceleration during the push-off phase from the flight time method could also affect the agreement with the force plate method, as variations in average force and velocity during the push-off phase are not necessarily related to jump height variations (17, 18, 52, 53)"

page 19, line 450

- Line 397. 'has' previously shown...

Thank you, "has" is added.

- Line 509. Values for analysis "are" used

This section is now removed to shorten the discussion as recommended.

- Line 510. the observation ... has, or the observations .... have..

This section is now removed to shorten the discussion as recommended.

- Line 511. that 'appears' when using...

This section is now removed to shorten the discussion as recommended.

- Line 517. less error for V0? Wouldn't this error be larger given that the relationship losses linearity particularly at values closer to v0?

Thank you, we agree that the sentence was unprecise, definitely meant to state larger errors for V0. However, this section is now removed to shorten the discussion as recommended.

- Line 534. push-off instead of push of.

Thank you, "push-off" is added.

- Line 547. The jumps…'were'

Thank you, "were" is added.

- Line 549. These variations…'are'.
Conclusions

Thank you, "are" is added.

- Line 55. Specify that this CV is between-session

Thank you, "between-session CV" is added.

Tables 3-5. Just in case it is possible. Could the tables be simplified assessing between-session reliability pooling all subjects together? Without dividing tests 1-2 and 3-4.

Thank you, we have considered this point and decided to change according to your suggestion for the figure (figure 3). However, in the table, readers can look at raw values and see whether the reliability changes with repeated testing experience.

Table 6. Please, specify what methods are being compared. What does the bias refer to in each line? CMJ vs leg press? CMJ vs SJ? Encoder vs flight time? Where is the comparison of leg press vs SJ flight time, for instance?

Thank you, we have changed the title to: "Table 5. Agreement and comparison for CMJ Force plate and SJ Force plate vs encoder, flight time and leg press measurements. "

Additionally, extra space and a line is added to make the table more readable.

Table 6. Footnote. Units are already specified in the table, no need to explain them in the footnote.

Thank you, the units for F_0, V_0, P_{max}, *and* S_{FV} *are removed.*

Table 6. Footnote. 'significant differences', or 'significantly different'? Significant different would be incorrect.

Thank you, we changed it to "significantly different**"**

Table 7. Please specify the units for F0, v0, Pmax and Sfv

Thank you, units are added.

Figure 2. tested instead of tester. Also, please, include the "n" always in capital or lowercase.

Thank you, we added “tested”, have changed to lower case “n” throughout the manuscript.

Figure 3. Panel B. The Y axis can be reduced (the maximum value is lower that 10-15). Also, the SWC is not discussed in the results section, and almost not discussed in the discussion section. I suggest either discussing this result in greater detail, or removing it. Were differences between days or between methods greater than the SWC?

Thank you for your comment. If we are to include SWC, we would like to have the same Y-axis at the CV% and SWC so that readers can easily visually compare these values. Similarly, the readers can compare the figure to the systematic bias and random error across methods presented in table 5. Additionally, we refer to the SWC figure at page 18, line 423.

“Additionally, the usefulness of a test is determined by the ability to detect changes in performance, more specifically by comparing the typical error (CV%) with SWC (Helland et al 2020 PeerJ). Indeed, the FV-variables obtained from the leg press apparatus showed superior signal-to-noise ratio compared to the other measurement methods in this study (Figure 3).”

Figure 5. If the aim is to assess the association between f-v parameters and performance measures, could this figure be simplified by removing the analyses on the left? The authors would just need to show the association between F0, v0, Pmax and Sfv with 1RM, CMJ, SJ, 10m sprint and 30m sprint, but there is no need to show the association between f0 leg press and f0 CMJ force plate, for instance.

Thank you for your comment, but we have removed the association between FV-parameters and performance measures from the manuscript as recommended. We have only included the figure for the FV-parameters as we believe this gives the reader a quick and visual impression of the results, although all the correlations might not be necessary. However, if the reviewer insist, we will remove this figure entirely.

Reviewer #2: Manuscript ID: PONE-D-20-29309

GENERAL COMMENTS: First, I would like to congratulate the authors on their effort. The study comprises a large sample given the elite level of the participants. The fact that several training centers participated on the research adds value to it. The aim of the study was to investigate the test-retest reliability and agreement across methods for assessing individual Force Velocity-profiles of the lower limbs in well-trained athletes. The research idea is interesting, and the conclusions may have important implications in current athlete profiling procedures. The investigation has a sound scientific background given the previously questioned reliability of the FVP parameters. However, some methodological aspects must be polished before I can recommend the publication of the study. I believe that some changes are necessary to strengthen the quality of the data reported and the conclusions. I do think that the data collected may positively impact the sport science field. The authors should interpret my comments as constructive.

Thank you for your comment and thorough job of reviewing the manuscript. We have answered all your comments below and show changes in the manuscript fil with the “track changes” function.

Abstract
Changes in the abstract will not be addressed prior to all the other issues within the

manuscript are solved.

Introduction
In general, the introduction tries to explain and introduce the research problem and is fairly successful. It is clear for the reader the need to conduct the present investigation.
Specific comments:

Line 59-60. "However, although several studies have evaluated the between-session reliability of FV-parameters". In general terms, what did these studies found? It would be interesting for the reader if the authors presented a bit more detail here.

Thank you for your comment, this sentence might have been a bit unprecise and is now modified. We have also included extra information regarding which equipment that have been used for the reliability analyses. Additionally, comments regarding previous findings are presented further down in the introduction.

However, although several studies have evaluated the within-session reliability of FV-variables (11-19), limited attention has been directed towards the between-session reliability of these FV-variables in athletes. Additionally, only encoders and the flight time calculation method have been used for measurements of between-session reliability of the FV-variables (12, 13, 19). Hence, reliability of other commonly used methods such as force plates and leg press devices are unknown (11-18).

page 2, line 68

"Interestingly, S_{FV} and V_0 have previously shown poorer reliability than F_0 and P_{max} in vertical jumping (11)."

page 3, line 87

Methods
The methods are interesting but that are some details that must be addressed.

Specific comments:
Line 103-105. "The first two (...) figure 1 & 2)". This sentence is somehow confusing. Based on the information presented in Figure 1, the authors should consider rephrasing as follows: "The first two testing timepoints were separated by ~1 week, before a period of 2~6 months. Then, the two-last testing timepoints were conducted also were separated by ~1 week (figure 1 & 2)."

Thank you, the sentence is modified accordingly:

"The first two testing timepoints were separated by ~1 week, before a period of 2~6 months. Then, the two last timepoints were also separated by ~1 week (Figure 1 and 2)."

page 4, line 106

Line 114. Please consider replacing "are" before "constant" with "were".

Thank you, "are" is replaced with "were"

Line 116. Please consider replacing "was" before "constant" with "were kept".

Thank you, "was" is replaced with "were"

Line 133. "national and elite level". What did the authors consider to be national and elite level? A better description of the criteria used to classify the athletes would be interesting for the reader.

Thank you, we have modified the sentence accordingly: "The competition level ranged from world class (Olympic medalist) to club level with the majority competing at the national and international level in their respective sport. "

page 5, line 140

Line 143. "hamstring and hip mobility". What do the authors mean by hamstring mobility? Please clarify.

Thank you, we have modified the sentence accordingly: "local muscle warm-up (hamstring- and hip mobility consisting of light dynamic stretches)"

page 6, line 155

Line 150-151. "for some weaker subjects, a protocol of approximately 5 loads up to 80% of bodyweight were used". Was there a specific criteria to determine this? Which athletes were considered as "weaker subjects"? And the 5 loads, were they standardized or individually determined? Please clarify.

Thank you, we have modified the sentence as follows: "In the aggregated sample, for some weaker subjects (i.e unable to jump with 80kg), a protocol of approximately 5 loads up to 80% of bodyweight were used. The increase in loads were then individually determined."

page 6, line 164

Line 161. Please consider replacing "was verbally forbidden" by "was not allowed".

Thank you, "was not allowed" is replaced with "was verbally forbidden"

Line 167. Please consider starting a new paragraph with "For the leg press,...". Otherwise, the paragraph will be too long and hard to follow.

Thank you, a new paragraph is started for the leg press.

Line 168-170. "(...) the FV-parameters were derived from a dedicated software based on a 10-repetition FV-test with incremental loading based on each athlete's 1RM load". This sentence is confusing. Please consider rephrasing.

Thank you, the sentence is modified accordingly: "The FV-variables were derived from a 10-repetition FV-test pre-programmed in the Keiser A420 software. To determine the loading range, the participants' 1RM was obtained at the familiarisation session for the main analysis, whereas the 1RM was individually estimated for the participants in the aggregated analysis."

page 7, line 184

Line 171. "~20% of 1RM". The 1RM load was previously determined? Or was the athlete's perceived 1RM used?

Thank you, see previous comment.

Line 177. It is spelled "heels" and not "heals". Please correct.

Thank you, typo is corrected.

Line 179. Please consider replacing "are" before "performed" with "is".

Thank you, “is” have been replaced with “are”

Line 183. Please consider starting a new paragraph with "Prior to the 30-m sprint, ...".

Thank you, we have removed this section due to recommendations from the reviewers.

Line 183. Did all participants perform the 30 m sprint test or just the athletes from running-based sports (e.g., soccer, handball or athletics)? I mean, did the weightlifters, ski-jumpers or badminton players also performed a 30-m sprint?
From what I understand one of the aims of the study is to "investigate the association between the FV-parameters obtained from vertical jumping and leg press with 1RM squat, jump height and 10 and 30m sprint time". I see no problem with investigating the association between the FV parameters and 1RM squat and jump height for the entire sample. However, when it comes to the sprint test, I am not sure that the analysis should include the weightlifters or sky-jumpers, for example. Do the authors consider that any meaningful practical application can be drawn from a ski-jumper's 30-m sprint performance?? If these athletes were included in the analysis for the sprint variables, I strongly recommend the authors to exclude them from this outcome. Also, I strongly suggest doing a sub-group with only the athletes from running-based sports (i.e., soccer, handball, ice-hockey, speed skating and possibly athletics - depending on the modality) and test the associations between FV parameters and 1RM, vertical jump and sprint outcomes. For the rest of the sample, I suggest testing the relationship between the FV parameters and only the 1RM and vertical jump (exclude sprint).
This will greatly strengthen the quality and, especially, the logic behind the study as it will provide meaningful data for practitioners, based on the specific physical performance variables from each sport without inducing them to perform unnecessary and “injury risk tests” (e.g., maximal sprint test for a weightlifter or a sky-jumper).

Thank you, we have removed this section due to recommendations from the reviewers.

Line 188. Please consider starting a new paragraph with "The 1RM back-squat...".

Thank you, we have removed this section due to recommendations from the reviewers.

Line 230. The fact that one force plate recorded at 200 Hz while other at 2000 Hz must be acknowledged as a limitation that may potentially affect data analysis and comparison between methods.

Thank you, this issue may not have been stated clearly. The agreement analysis is only from one force plate, whereas the aggregated analysis includes two force plates. To make this clearer we have added the following:

"Therefore, the main analysis in this study are performed on the subjects tested under all methods (reliability and agreement), with an additional aggregated analysis including all subjects with varying sample size across methods (only reliability analysis)."

page 4, line 115

"Additionally, for the analysis for agreement the force plate sampled at 200 Hz compared to 1000 Hz used previously (15) which might influence the findings. For the aggregated reliability analysis, both 200 Hz and 2000 Hz force plates were used, and we would argue that the findings of reliability seem independent of sampling frequency. "

page 22, line 535

Results
The figures are well designed and facilitate the interpretation of the data.
Line 291. As stated before, I strongly recommend the authors to exclude the athletes from non-running based sports from the sprint analysis to strengthen the conclusion and ecological validity of the data reported in the study.

Thank you, the associations are now removed due to recommendations from the reviewers.

Discussion
Overall, the discussion is well written, and the authors do a good job comparing the results obtained with previous research. They present a sound reasoning for their findings, supporting their data adequately based on previously published literature. However, the section is too lengthy and would greatly benefit if the authors were able to be more concise with their writing and reduce the word count. Some of the paragraphs repeat information, which makes this section hard to follow at times. I strongly recommend the authors to invest time re-arranging the discussion because I do believe that the paper is interesting for the sport science community and has the potential to be published and impact current athlete profiling practices.

Thank you for your comment. The discussion section is reduced from 3031 words to approximately 2300 words

Specific comments:

Line 306-334. This paragraph is too long. I recommend the authors to shorten it or divide it into multiple paragraphs.

Thank you, this section have been divided into two parts.

Line 334. All tables should be presented in the Results section. I recommend the authors to move Table 8 into the mentioned section.

Thank you, table 8 is moved to the results section

Line 353. According to the table presented in Table 7, the Pmax obtained in the SJ was higher than the CMJ when using the encoder. However, in the other methods, the Pmax was higher in the CMJ (as it would be expected). The authors must explain these contradicting results in this paragraph.

Thank you for noticing this issue! We found an error in the plotting of the numbers which is now corrected. The pmax for CMJ encoder is 1906 and for SJ encoder 1652. Really sorry for this, but thank you again for noticing the unfortunate error.

Line 378-385. The information presented in this paragraph have been previously presented in the discussion section (Line 319, 322-327). The authors should consider removing this part of the text to avoid repetition and improve the "fluidity" of the discussion.

We agree and this paragraph is now removed.

Line 397. Please consider replacing "have" before "previously" with "has".

Thank you, "have" is used instead of "has"

Line 407-437. This paragraph is too long. I recommend the authors to shorten it or divide it into multiple paragraphs.

Thank you, we have divided the section into two paragraphs.

Line 479. "All FV-variables depend on the measurement condition, including equipment, exercise type, resistance modality and push-off distance." What are the practical applications from this? I believe the discussion could be shortened and provide a more "applied" perspective.

Thank you, the discussion is shortened as recommended including removing the entire paragraph containing the discussion regarding FV-parameter associations. Additionally, remarks regarding the applied perspective is added under "Conclusions and Practical applications":

"Efforts should be made to either reduce the variation in jumping performance or assessing loads closer to the FV-intercept. Increasing the loading range can be achieved through using alternative exercises such as a leg press exercise. Reducing the variation in jumping performance could possibly be achieved through additional practice attempts, and attention should be given to the depth of the squatting motion during the vertical jumps. "
page 22, line 545

Line 483-521. This paragraph is too long. I recommend the authors to shorten it or divide it into multiple paragraphs.

Thank you, this is removed based on the reviewer's suggestion.

Line 509. Please consider replacing "is" before "used" with "are".

Thank you, this is removed based on the reviewer's suggestion.

Line 532. Strengths and limitations. Another important limitation is related to the fact that, for the aggregated analysis, force plates with different sampling frequencies were used. This must be acknowledged in the manuscript and its implications for the results must be briefly addressed (one sentence would suffice).

Thank you, this issue might not have been stated clearly. The agreement analysis is only from one force plate, whereas the aggregated analysis includes two force plates. To make this clearer we have added the following:

“Therefore, the main analysis in this study are performed on the participants tested under all methods (reliability and agreement), with an additional aggregated analysis including all participants with varying sample size across methods (only reliability analysis).”

page 4, line 116

“Additionally, for the analysis for agreement the force plate sampled at 200 Hz compared to 1000 Hz used previously (15) which might influence the findings. For the aggregated reliability analysis, both 200 Hz and 2000 Hz force plates were used, and we would argue that the findings of reliability seem independent of sampling frequency.”

page 22, line 535

Line 526. The argument used regarding the "ecological validity" of the study is greatly affected by the fact that athletes whose training regimens do not usually incorporate linear sprint actions (e.g., weightlifting or badminton), where tested for 30-m sprint performance. For this reason, the authors should re-consider the analysis made. The data presented herein is really interesting and address important aspects related to athlete profiling. I consider that publishing such data would be important for the sport science community. It would be relevant, for example, to include some discussion related to this issue, emphasizing that athletes from non-running/sprint-based sports should not perform sprint-based tests, as this is “not rational from a logical standpoint”. Therefore, I strongly advise making the previously mentioned adjustments as it would greatly improve the quality of the data.

Thank you, as recommended this analysis is removed in this manuscript and we will probably publish these results as a separate analysis. We appreciate your comment and will consider your point in the second analysis.

Line 574. Please consider replacing "was" before "performed" with "were".

Thank you, “were” is now used.

547-549."The jumps in this study were performed with free weights where it was difficult to accurately standardize the center of mass of the jumps using only thigh depth or knee angle as a reference." Although I understand what the authors mean here, I am not sure if performing the exercises with free weights is an actual limitation of the study. Most athletes train with free-weights, so that increases the "ecological validity" of the data reported. Moreover, it is really important for coaches to understand that calculating FV-parameters using free weights affects test-retest reliability due to the reasons mentioned throughout the manuscript. It is not a limitation, but rather an “unavoidable” phenomenon that occurs is real-world training settings.

Thank you, we completely agree and have added this to the end of the sentence: “Nevertheless, the use of free weights increases the ecological validity of the study as this is commonly used by athletes.”

Conclusions and practical applications
The conclusions seem appropriate and based on the results obtained.

Thank you
