# Supplementary material for: Force-velocity profiling in athletes: Reliability and agreement across methods
Source: PLoS One. 2021 Feb 1;16(2):e0245791. doi: 10.1371/journal.pone.0245791 (PMC7850492; doi:10.1371/journal.pone.0245791)
Supplement: Attachment — Submitted filename: Response to Reviewers.docx [file pone.0245791.s004.docx]

We would like to thank the reviewers for reviewing this manuscript. The reviewer comments are copied in below with **bold text**, and the changes made in the revised version of the manuscript are in red and explained directly below.

**Reviewer #1: I would like to thank the authors for addressing most of my previous comments. I think the manuscript would benefit from revising some minor typos (please, see some examples below), but is overall acceptable for publication. It would be good if an English native speaker revises the writing (given that PLOS One does not copyedit accepted manuscripts).**

**Thank you, we have read through several times to correct typos (se revisions in the file “revised manuscript”). Additionally, the typos you pointed out is corrected.

Last line of abstract and conclusion: “as well as OF THE differences across measurement methods…” The of is missing.**

**Thank you, ”of” is added. (Line 47)**

**Introduction. Line 63. Controversy “exists”. The “s” is missing.**

**Thank you, ”s” is added. (Line 63)**

**Introduction. Line 67. Reference number 19 assessed between-session reliability, not within-session reliability.**

**Thank you, reference 19 is removed from this sentence (Line 67)**

**Methods, Line 163-164: A protocol of…”was used”. Is the verb referring to “protocol” or to “the loads”?**

**Thank you, ”was used” is added. (Line 164)**

**Methods, Line 164: The increase in loads “was” then individually determined, or loads “were” then individually determined.**

**Thank you, ”was” is added. (Line 164)**

**Methods, line 186-191: Please be consistent with the verb tense (past simple), i.e., "was gradually increased..."**

**Thank you, this is now fixed. “The test started”, “the load was gradually increased”, “The rest period between attempts got longer”, “The rest period between attempts was”, (line 186-191)**

**Results, line 277: the typical error...was, or the typical errorS (in plural)...were**

**Thank you, ”was” is added. (Line 277)**

**Discussion, Line 366 The correct citation would be Valenzuela et al. (Pedro L. is the name, not the surname). Also applicable in the reference list.**

**Thank you, “Valenzuela” is added (Line 366) (and updated in reference list)**

**Discussion, Line 424: No need to mention the journal (PeerJ) here. Please, revise the citation.**

**Thank you, the reference is now fixed (Line 424)**

**Reviewer #2: Manuscript: PONE-D-20-29309R1

GENERAL COMMENTS: I would like to congratulate the authors for their effort to address the points raised. I believe that the quality of the paper has greatly improved to the level expected for its publication. The authors have reformulated their discussion which allows for a better understanding of the phenomenon being studied. This manuscript adds to the recent and compelling evidence questioning the reliability of the FV variables which has great implications for coaches and sport scientists. I am happy to endorse the publication of the manuscript. However, the authors should adjust the following minor details:

Tables 3 and 4. Tables 3 and 4. For consistency concerning the rest of the manuscript, please report the ICC values as "0.81" instead of ".81".**

**Thank you, these tables are now updated

Line 366 (consider the line numbers of the manuscript version without marked changes). Please note, that the author is "Valenzuela et al. [19]" and not "Pedro L. et al." Also, this reference should be corrected in the reference list.**

**Thank you, “Valenzuela” is added (Line 366) (and updated in reference list)**

**Line 424. The reference “(Helland et al 2020 PeerJ)” must be corrected so per journal style. In addition, I was not able to find this reference in the reference list.**

**Thank you, the reference is now fixed (Line 424)**

**Line 529. Please replace "This variations" with "These variations".**

**Thank you, ”These” is added. (Line 529)**
